# Pruritic, erythematous, arcuate, and serpiginous smooth plaques

**DOI:** 10.1016/j.jdcr.2021.04.030

**Published:** 2021-05-06

**Authors:** Kimberly Blain, Elliot Blue, Jamie Zussman, Jennie T. Clarke

**Affiliations:** a Department of Dermatology, University of Utah, Salt Lake City, Utah; b Department of Internal Medicine, Medical College of Georgia at Augusta University, Augusta, Georgia

**Keywords:** pruritic, serpiginous, syphilis, the great imitator, treponema pallidum, FTA-ABS, Fluorescent treponemal antibody absorption.

A 59-year-old man with diabetes mellitus, hypertension, coronary artery disease, and gout presented to the dermatology clinic with an 18-month history of a pruritic widespread rash (Fig 1). Dull, erythematous, arcuate, and serpiginous smooth plaques, involving the chest, abdomen, arms, and back with a few scattered, scaly, erythematous plaques intermixed were observed. Episodes of the rash lasted for several months at a time, had no clear triggers, and resolved spontaneously. Previous treatments included triamcinolone 0.1% cream, fluconazole 150 mg weekly for 4 weeks, and terbinafine 1% cream without improvement. He denied sexual activity in the last year and a half, and he was HIV negative. Biopsies were obtained, and hematoxylin-eosin and immunohistochemical staining were performed (Fig 2 [original magnification ×100] and Fig 3 [original magnification ×400]).

**Fig 1:**
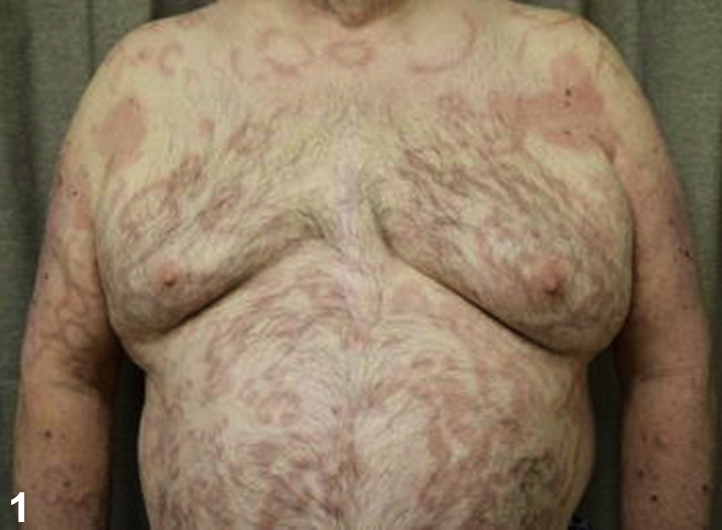

**Fig 2:**
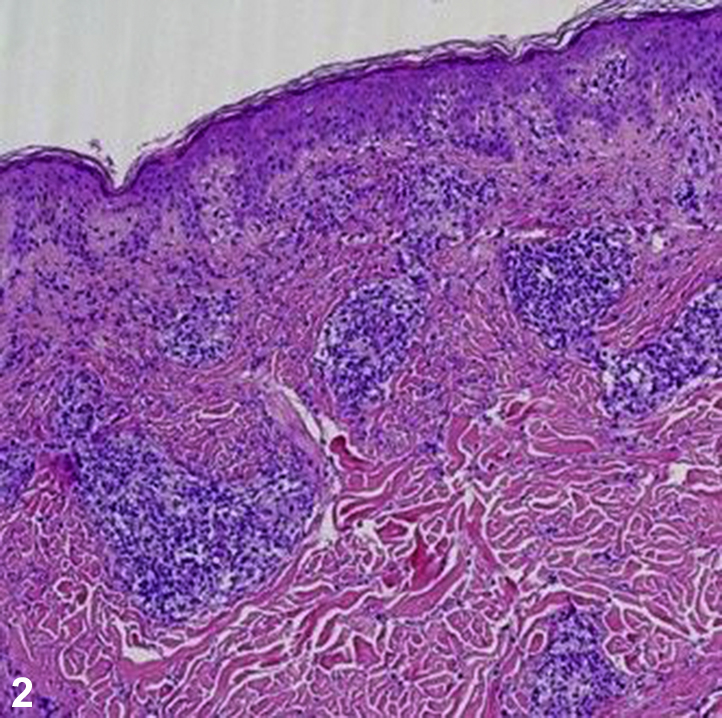

**Fig 3:**
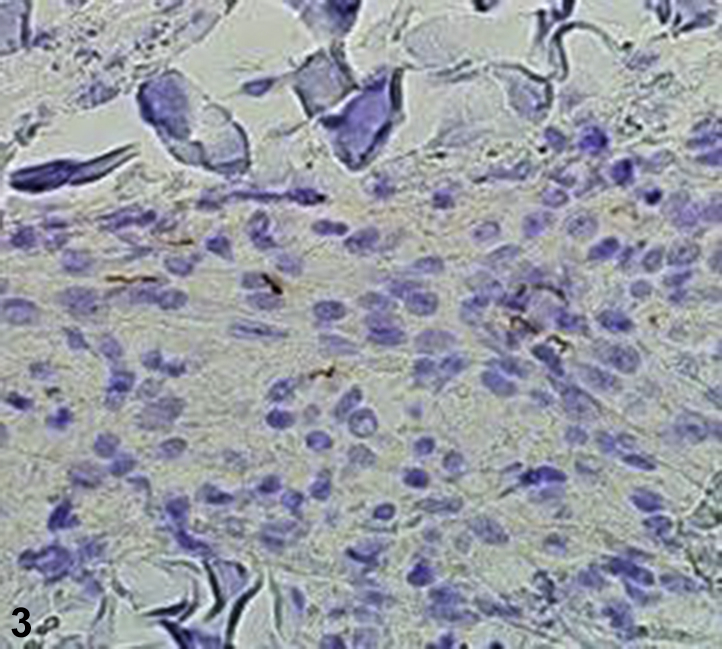

**Question 1: What is the most likely diagnosis?**

A. Mycosis fungoides
B. Disseminated granuloma annulare
C. Secondary syphilis
D. Erythema gyratum repens
E. Sarcoidosis

**Answers:**

A. Mycosis fungoides – Incorrect. Mycosis fungoides is the most common type of cutaneous T-cell lymphoma and classically presents with scaly erythematous patches and plaques with a predilection for the trunk, specifically the buttocks. Mycosis fungoides is also notorious for a variety of clinical presentations. This diagnosis was excluded by histopathology.
B. Disseminated granuloma annulare – Incorrect. Disseminated granuloma annulare is a self-limiting benign skin condition presenting with symmetric annular or arciform plaques without surface change as well as discrete skin-colored papules. Disseminated granuloma annulare is not common and tends to be recalcitrant to therapy. Clinically, this diagnosis was favored until the histopathology was reviewed.
C. Secondary syphilis – Correct. Secondary syphilis is nicknamed “the great mimicker” because it exhibits a variety of cutaneous morphologies and histological findings.^1^ While cutaneous findings vary, the most common clinical presentation is a widespread, nonpruritic, copper-colored, papulosquamous eruption involving the palms and soles.^2^ Histopathology demonstrates mild vacuolar interface alteration with superficial and deep perivascular lymphohistiocytic inflammation. Immunohistochemistry positive for spirochetes (*arrow*) solidified the diagnosis (Fig 4).

**Fig 4:**
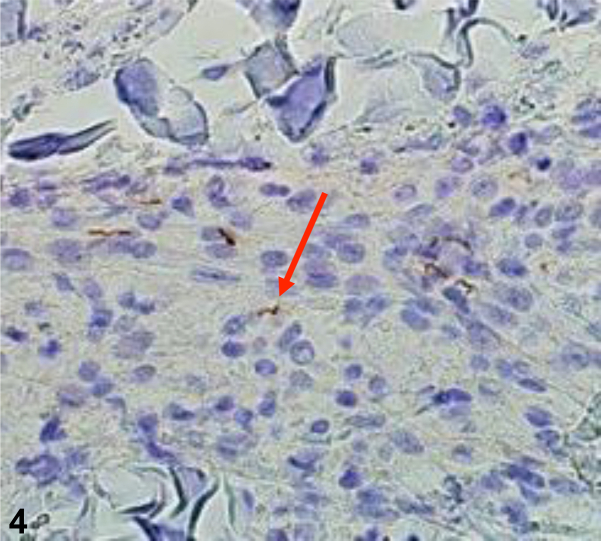
D. Erythema gyratum repens – Incorrect. Patients presenting with erythema gyratum repens, which is a rare, transient, paraneoplastic, figurate erythema, exhibit multiple, annular, or polycyclic patches or plaques with overlying scale. This patient's rash had been fixed for several months, making a diagnosis of erythema gyratum repens unlikely.
E. Sarcoidosis – Incorrect. Cutaneous manifestations of sarcoidosis occur in up to 25 percent of the patients and may present with a variety of morphologies, including papules, nodules, plaques, and infiltrated scars. The classic histopathologic findings are noncaseating granulomas with sparse peripheral lymphocytic infiltrates, which were not present in this patient.

**Question 2: What is the appropriate treatment for this patient?**

A. Procaine penicillin 50,000 U/kg intramuscularly daily for 10 days
B. Narrow-band ultraviolet B phototherapy
C. Benzathine penicillin 2.4 million units intramuscularly once
D. Hydroxychloroquine 200-400 mg daily for at least 3 months
E. Doxycycline 100 mg twice a day for 14 days

**Answers:**

A. Procaine penicillin 50,000 U/kg intramuscularly daily for 10 days – Incorrect. Procaine penicillin is the treatment of choice for congenital syphilis.
B. Narrow-band ultraviolet B phototherapy – Incorrect. In patients with mycosis fungicides involving 10% or more of the total skin surface with no lymph node involvement, treatment with topical corticosteroids, topical nitrogen mustard, or narrow-band ultraviolet B phototherapy is recommended.
C. Benzathine penicillin 2.4 million units intramuscularly once – Correct. Typically, cutaneous manifestations of syphilis resolve within 3 months, and the patient enters the early latent phase. However, 25% of the patients will have relapses of secondary syphilis manifestations usually in the first 1-2 years. First-line therapy for secondary syphilis is benzathine penicillin 2.4 million units intramuscularly once.^3^
D. Hydroxychloroquine 200-400 mg daily for at least 3 months – Incorrect. In patients with sarcoidosis with extensive cutaneous involvement, hydroxychloroquine and other antimalarial agents have shown therapeutic benefit.
E. Doxycycline 100 mg twice a day for 14 days – Incorrect. In non-pregnant patients with a legitimate penicillin allergy who cannot be desensitized, doxycycline is the first-line alternative agent in secondary syphilis.

**Question 3: Which lab test can be used to monitor treatment response?**

A. Rapid plasma reagin
B. Fluorescent treponemal antibody absorption (FTA-ABS)
C. Angiotensin-converting enzyme levels
D. *Treponema pallidum* enzyme immunoassay (IgG)
E. Chemiluminescence immunoassay

**Answers:**

A. Rapid plasma regain – Correct. Nontreponemal tests such as rapid plasma regain, venereal disease research laboratory, and toluidine red unheated serum tests are traditionally used as initial screening tests but also as the main way of monitoring a patient's response to treatment due to their quantitative results.^4^ Successful therapy will be indicated by a decline in antibody titers.
B. FTA-ABS – Incorrect. FTA-ABS is a qualitative treponemal test, thus providing no useful tracking of treatment response. The FTA-ABS assay is thought to be most sensitive in primary syphilis.
C. Angiotensin-converting enzyme levels – Incorrect. Angiotensin-converting enzyme levels correlate with disease severity and activity in response to treatment in sarcoidosis, not syphilis.
D. *Treponema pallidum* enzyme immunoassay (IgG) – Incorrect. The *T. pallidum* enzyme immunoassay is also a treponemal test that has become a favorite of large laboratories as a screening test due to its lower cost compared with the others.
E. Chemiluminescence immunoassay – Incorrect. The chemiluminescence immunoassay is also a treponemal test. All treponemal tests are based upon the detection of antibodies directed against specific treponemal antigens and thus tend to be more specific than nontreponemal tests.

## Conflicts of interest

None declared.
